# The granulation tissue preservation technique in regenerative periodontal surgery—a randomized controlled clinical trial

**DOI:** 10.1002/cre2.532

**Published:** 2022-01-11

**Authors:** Knut Adam, Hüsamettin Günay, Bernhard Vaske, Marco Flohr, Ingmar Staufenbiel

**Affiliations:** ^1^ Department of Conservative Dentistry Periodontology and Preventive Dentistry, Hannover Medical School Hannover Germany; ^2^ Institute of Biostatistics, Hannover Medical School Hannover Germany

**Keywords:** granulation tissue, infrabony, osseous defect, periodontitis, regeneration

## Abstract

**Objectives:**

To investigate if the application of the granulation tissue preservation technique (GTPT) in regenerative therapy of infrabony periodontal defects results in more clinical attachment level (CAL) gain and more radiographic bone gain (RBG) than the conventional resective approach 12 months after surgery.

**Materials and methods:**

Forty patients exhibiting at least one infrabony defect with a probing pocket depth (PPD) ≥6 mm and a radiographic infrabony component (INFRA_X‐ray_) ≥3 mm were randomly treated with the GTPT (test group) or the double‐flap approach with resection of the defect‐filling granulation tissue (control group). Enamel matrix derivatives were applied in both groups. Clinical and radiographic parameters were recorded at baseline (t0), 6 months (t1), and 12 months (t2) after surgery. The primary outcome variable was CAL gain between t0 and t2.

**Results:**

When all patients were considered, ΔCAL_t0–t2_ did not differ significantly between the two groups (*p* = .160). Significant PPD reduction (test group: 4.38 ± 1.36 mm; control group: 4.06 ± 2.38 mm), CAL gain (test group: 3.75 ± 1.24 mm; control group: 2.88 ± 2.09 mm), and RBG (test group: 3.06 ± 1.74 mm; control group: 3.27 ± 2.19 mm) were achieved at t2 in both groups. Using multivariate linear regression, PPD_t0_ and group were identified as variables with the greatest influence on ΔCAL_t0–t2_. PPD_t0_ and INFRA_X‐ray_ were identified as variables with the greatest influence on RBG_t0–t2_. Patients with a defect angle >22° showed significantly more CAL gain in the test group (t0–t1: 3.08 ± 1.38 mm; t0–t2: 3.62 ± 0.96 mm) than in the control group (t0–t1: 1.77 ± 1.54 mm; t0–t2: 2.18 ± 1.83 mm).

**Conclusions:**

Regarding all patients, the study failed to show significant differences between the test and control groups. However, the GTPT appears to lead to more CAL gain in noncontaining infrabony defects.

## 1 BACKGROUND

Minimally invasive surgical techniques have been developed to limit the extent of the surgical area, to achieve a stable primary wound closure, and thus to avoid failures in wound healing particularly in the area of the interdental papilla (Cortellini & Tonetti, 2007, 2009; Harrel, 1998; Harrel & Rees, 1995; Trombelli et al., 2009). All these flap designs have in common that the defect‐filling granulation tissue is resected and discarded. However, advanced infrabony defects are often not limited to the interdental space but extend to the oral and buccal sites. In these cases, it is not sufficient to prepare only one flap, but a double flap approach must be used to ensure sufficient visibility of the root surface to be instrumented. In addition, advanced complex defects usually lack soft tissue support, which is essential for the success of the regenerative procedure. Therefore, bone substitutes are frequently used to fill the space previously occupied by granulation tissue and thus to preserve space for regeneration (Kao et al., 2015; Reynolds et al., 2003). It has been shown for different graft materials that the space created for regeneration leads to new bone formation only to a limited extent (Stavropoulos et al., 2003). Furthermore, graft materials carry the risk of microbial contamination compromising the treatment outcome.

Recently, we introduced the granulation tissue preservation technique (GTPT) for regenerative therapy of infrabony periodontal and peri‐implant defects (Günay et al., 2013, 2019). Preservation of the defect‐filling granulation tissue is hypothesized to serve as soft tissue support, to make the use of bone substitutes dispensable, and to enable increased wound stability particularly in the area of the interdental papilla. Moreover, the existing vascular network and the precursor cells contained in the granulation tissue can be preserved and subsequently promote wound healing. In vitro and in vivo studies have shown that cells with properties of mesenchymal stem cells reside in both inflamed periodontal and peri‐implant tissues (Adam et al., 2019, 2020; Gousopoulou et al., 2021; Park et al., 2011). From this point of view, it seems to make sense to preserve the defect‐filling granulation tissue in regenerative periodontal surgery. Preservation of granulation tissue was first described by Lindhe and Nyman (1985). They already stated that granulation tissue removal during access flap surgery is not a mandatory measure for creating suitable conditions for the proper healing of periodontal tissues.

The aim of the present study was to compare the GTPT with the conventional double‐flap approach, in which the defect‐filling granulation tissue is resected. Since soft tissue collapse was to be expected particularly in defects with missing bony support, the statistical examination focused on noncontaining defects defined by large radiographic defect angles. The hypothesis of the present study was that infrabony periodontal defects treated with the GTPT would result in more clinical attachment level (CAL) gain and more radiographic bone gain (RBG) than those treated with the conventional approach.

## 2 METHODS

### 2.1 Experimental design

**Figure 1:**
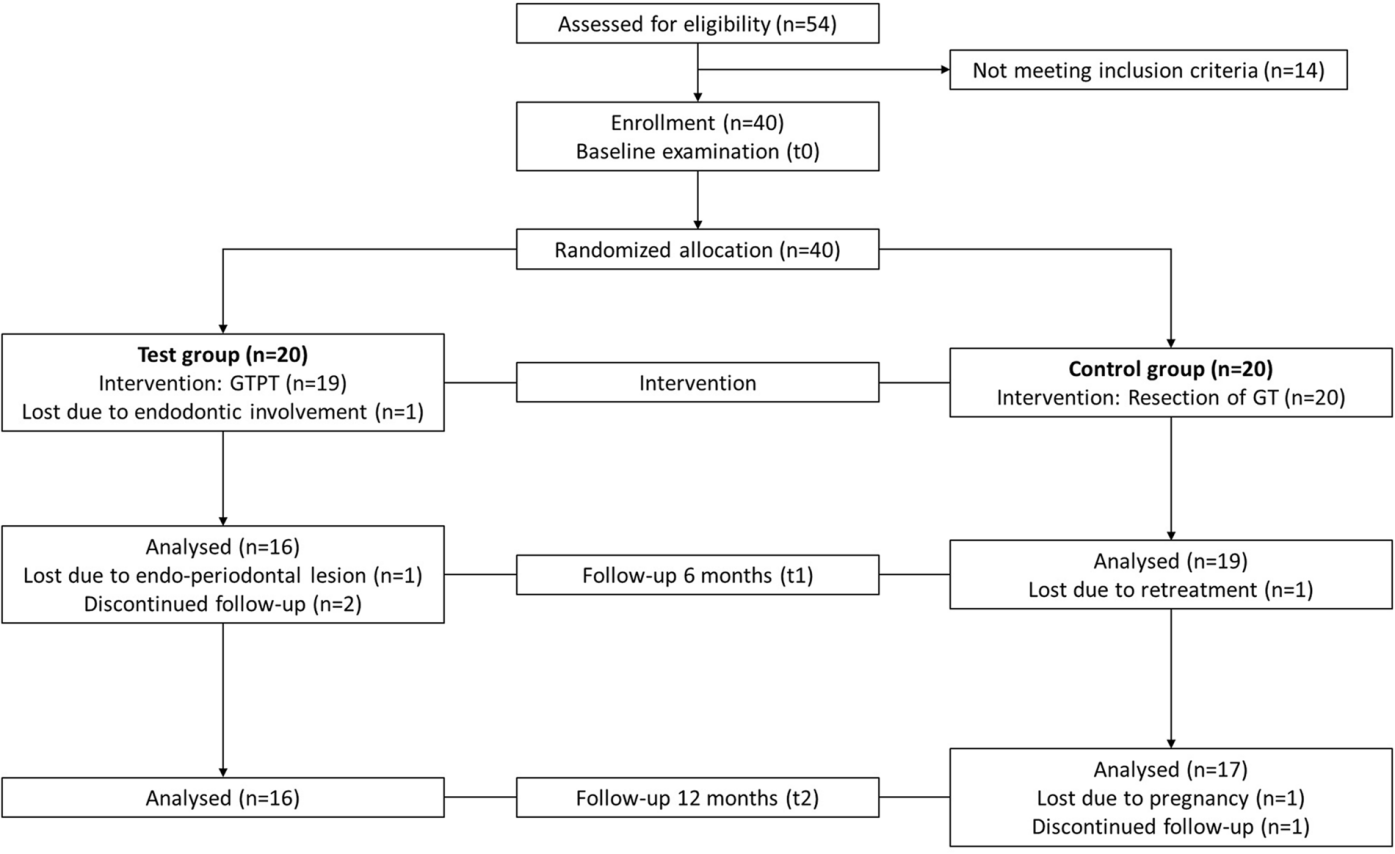
Study flow diagram

The present clinical trial was performed in the Department of Conservative Dentistry, Periodontology, and Preventive Dentistry of Hannover Medical School (MHH) and had a prospective, randomized, controlled, and double‐blinded (patients, investigator) design. In total, 40 patients with 40 deep infrabony periodontal defects received regenerative periodontal surgery using the GTPT (test group; *n* = 20) or the conventional double flap approach with resection of the defect‐filling granulation tissue (control group; *n* = 20). In both groups, enamel matrix derivatives (EMDs) were used as bioactive molecules to promote periodontal regeneration. The clinical and radiographic outcomes of both groups were longitudinally followed for 12 months (Figure 1). The study was conducted in accordance with the Declaration of Helsinki as revised in 2013. The Institutional Review Board (Ethical Committee of MHH) approved the study protocol (ethics vote no. 5556) and all participants signed informed consent.

### 2.2 Patient and defect eligibility

Systemically healthy individuals presenting with advanced periodontitis (Stage III or IV) and at least one isolated deep, mostly interproximal infrabony defect were considered appropriate for this trial. The inclusion criteria were

(1) Probing pocket depth (PPD) ≥ 6 mm
(2) Radiographic infrabony component (INFRA_X‐ray_) ≥ 3 mm
(3) Positive response to sensitivity test with refrigerant spray
(4) Hygiene index (HI, see below) ≥ 40% recorded during the first step of periodontal therapy

When more than one eligible defect was available, the one with the largest INFRA_X‐ray_ (see below) was selected. The exclusion criteria comprised:

(1) Heavy smokers (>10 cigarettes daily)
(2) Pregnancy
(3) Breastfeeding
(4) Intake of antibiotics and/or nonsteroidal antirheumatic drugs within the previous 3 months
(5) Systemic diseases with known impact on periodontal health

Before baseline examination, all patients received the first and second steps of periodontal therapy consisting of supragingival dental biofilm control, oral hygiene instructions, professional mechanical plaque removal, elimination of possible plaque‐retentive factors, and subgingival instrumentation (Sanz et al., 2020). Six weeks after completion of the second step of periodontal therapy, clinical and radiographic baseline examinations were conducted.

### 2.3 Clinical and radiographic parameters were recorded at baseline (t0) and 6‐months (t1) and 12‐months (t2) follow‐up visits

The clinical parameters recorded at baseline (t0), 6 months (t1), and 12 months (t2) after surgery included bleeding on probing (BOP, Ainamo & Bay, 1975), full mouth bleeding score (FMBS), HI, PPD, recession depth (RED), and CAL. BOP, FMBS, PPD, RED, and CAL were assessed at six sites per tooth (mesiobuccal, buccal, distobuccal, mesio‐oral, oral, disto‐oral) using a WHO periodontal probe. BOP was assessed dichotomously (yes/no) and subsequently used to calculate the FMBS (Cortellini et al., 1993). The FMBS was calculated using the formula: sum of bleeding sites/sum of all sites × 100 in percent. The HI is a modification of the plaque control record (O'Leary et al., 1972) and was used to assess the quality of oral hygiene measures at home. For this purpose, all teeth were stained using a plaque revelator (Mira‐2‐Ton, Hager & Werken) and evaluated dichotomously (yes/no) at four sites per tooth (mesial, distal, buccal, oral). The HI was calculated using the formula: sum of plaque‐free sites/sum of all sites × 100 in percent. The simplified composite outcome measure (COM) was used to evaluate the success of regenerative periodontal surgery (Trombelli et al., 2020). Briefly, the changes in PPD and CAL between t0 and t2 were assigned to the following categories in a 2 × 2‐frequency table:

(1) CAL gain ≥ 3 mm, residual PPD ≤ 4 mm (treatment success),
(2) CAL gain ≥ 3 mm, residual PPD > 4 mm,
(3) CAL gain < 3 mm, residual PPD ≤ 4 mm,
(4) CAL gain < 3 mm, residual PPD >4 mm (treatment failure).

**Figure 2:**
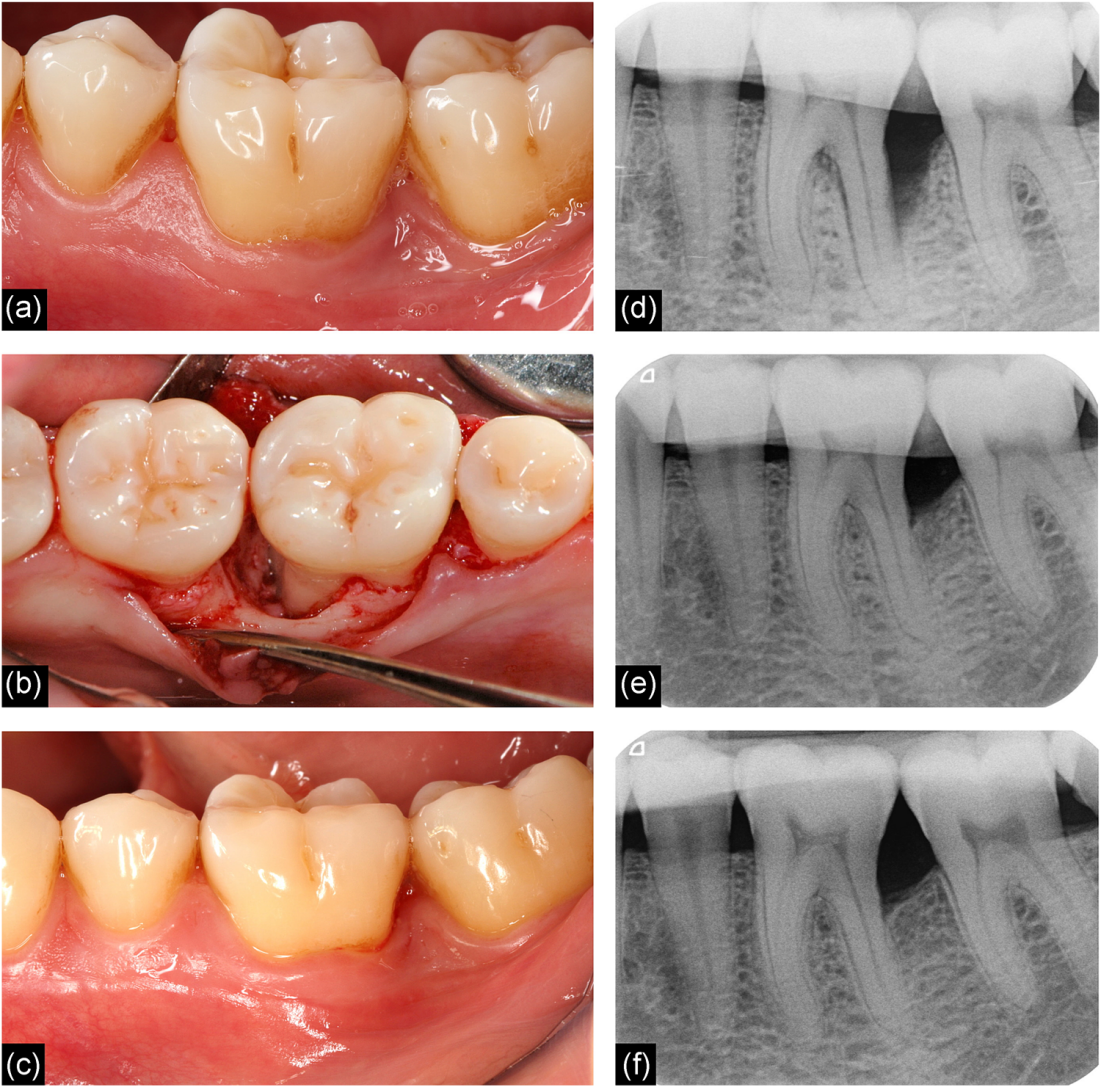
Application of the granulation tissue preservation technique on tooth 36. Clinical view: (a) before surgery, (b) after mobilization of the mucoperiosteal flap and instrumentation of the defect‐related root surface, and (c) 12 months after surgery. Note the completely preserved height of the interdental papilla. (d) Radiographic view of the infrabony defect using an individualized X‐ray film holder. Significant radiographic bone gain was observed (e) 6 months and (f) 12 months after surgery

X‐rays were taken at t0, t1, and t2. X‐ray film holders (Super‐Bite, Kerr) were individualized using addition‐curing silicone (Silagum‐Putty, DMG) to warrant a reproducible beam path and best possible comparability of the radiographic images (Figure 2). The following distances were measured at sites affected by the infrabony defect using a software program for dental imaging (byzzKlinik, orangedental):

(1) Distance from the cementoenamel junction to the bottom of the defect (CEJ‐BD_X‐ray_)
(2) Distance from the cementoenamel junction to the root tip (CEJ‐RT_X‐ray_)

The RBG at t1 and t2 was calculated using the formula:

RBG_t0–tx_ = (CEJ‐BD_X‐ray_)_t0_ – ([CEJ‐BD_X‐ray_]_tx_ × [CEJ‐RT_X‐ray_]_t0_/[CEJ‐RT_X‐ray_]_tx_).

Besides this, (INFRA_X‐ray_ = distance from the bone crest to the bottom of the defect) and the radiographic defect angle were determined using the byzzKlinik software.

### 2.4 Surgical procedure

**Figure 3:**
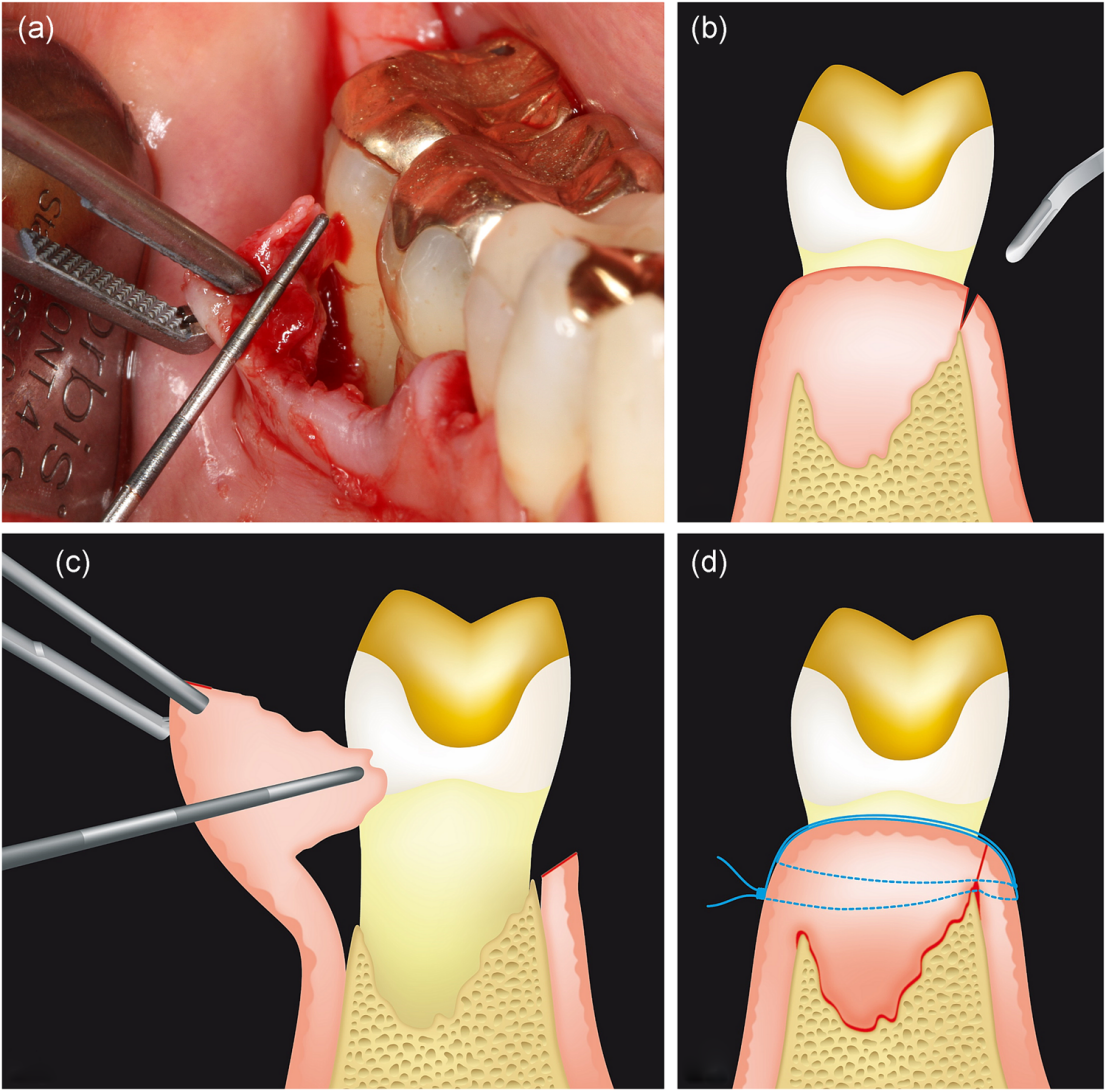
Schematic illustration of the granulation tissue preservation technique. (a) Representative patient case with an infrabony periodontal defect at the distal site of tooth 46. Note the amount of granulation tissue preserved at the buccal mucoperiosteal flap. (b) Ideal positioning of the interdental incision on the bone crest. (c) Complete mobilization of the defect‐filling granulation tissue remaining attached to the mucoperiosteal flap. (d) Repositioning of the mucoperiosteal flap and wound closure with suture

Analgesia was achieved through infiltration or block anesthesia using an epinephrine‐containing local anesthetic (Ultracain D‐S forte, Sanofi‐Aventis). Circumferential, strictly intrasulcular incisions were conducted at the defect‐related teeth using a microsurgical blade (Micro Miniature Blade #6962, Surgistar, Vista). An oblique, z‐shaped incision was applied at the interdental space to connect the intrasulcular incisions of two adjacent teeth. The localization of the interdental incision was dependent on the defect morphology (residual buccal and/or oral bone wall) and the width of the interdental space. In interdental spaces with a width ≥ 2 mm and a preserved buccal and/or oral bone wall, the interdental incision was ideally placed on the marginal bone crest (Figure 3), which sometimes had to be localized by bone sounding. Afterward, the mucoperiosteal flap was first prepared on the side of the alveolar process, where the marginal bone crest had been preserved. This procedure allowed the granulation tissue to be separated from the root surface and the bony defect walls under direct vision. For interdental spaces with a width < 2 mm or those with missing buccal and oral bone walls (one‐wall defects), the interdental incision was placed centrally in the area of the proximal contact. In this procedure, the defect‐filling granulation tissue was divided into a buccal and an oral portion (Video Clip S1). Generally, mobilization of the interdental soft tissues (including granulation tissue) was performed with sharp, microsurgical instruments (e.g., periotome PT1X, Goldman‐Fox Knife KGF11X, Periosteal Elevator PH26M; all from Hu‐Friedy) and under constant contact with the bony defect walls. The mucoperiosteal flaps were mobilized only to the extent that sufficient space was available for instrumentation of the defect‐related root surface(s). This was performed with sonically driven instruments (SONICflex, KaVo Dental) and Gracey mini curettes (American Eagle Instruments, Young Innovations Europe). After conscientious removal of the microbial deposits, the clinical infrabony component (INFRA) was measured. Subsequently, the regenerative procedure was applied. The root surface was conditioned for 2 min with 24% EDTA (PrefGel®, Straumann) and irrigated with sterile isotonic sodium chloride solution. After careful air‐drying, EMDs (Emdogain®, Straumann) were applied onto the root surface(s). The mucoperiosteal flaps were repositioned and fixed at the base of the interdental papilla with interrupted sutures (GORE‐TEX Suture CV‐6, W. L. Gore & Associates). In the test group, special focus was placed on the exact repositioning of the granulation tissue into its original position within the infrabony defect (Figure 3). In the control group, the granulation tissue was completely resected before the interdental papillae were repositioned and fixed. Finally, the operating area was gently compressed with saline‐soaked gauze for 1 min.

## 3 POSTOPERATIVE CARE

Patients were instructed to spare the surgical area, to refrain from mechanical plaque control, and to use instead a mouth rinse containing 0.2% chlorhexidine digluconate twice daily. Patients attended weekly control sessions during the first 3 weeks. At each visit, the surgical area was carefully cleaned and epithelial wound healing of the interdental papilla was assessed using the early healing index (Wachtel et al., 2003). The sutures were removed 2 weeks after surgery. After this initial 3‐week wound healing phase, patients were again allowed to brush their teeth with a very soft toothbrush. The use of interdental brushes was permitted depending on the progress of the papillary soft tissue healing. Supportive periodontal therapy was given at 3‐monthly intervals. This included professional supra‐ and subgingival tooth cleaning and remotivation and re‐instruction of the patients to maintain the best possible oral hygiene.

### 3.1 Statistical analysis

The Institute of Biostatistics (MHH) conducted the sample size calculation (nQuery Advisor 7.0) and randomized patient allocation. The difference of CAL between t0 and t2 (ΔCAL_t0–t2_) was defined as the primary outcome variable. A mean difference of 1.5 mm was expected between the test and control groups. The sample size calculation assumed that there was an unrelated problem, a Type I error of 5% (two‐sided), and a standard deviation of 1.5 mm. For this setting, a power of 92% was calculated for 20 patients per group. The values used for the sample size calculation were based on data obtained from studies using the modified Widman flap as resective approach (Heitz‐Mayfield et al., 2002) and using the simplified papilla preservation flap as a tissue‐preserving approach (Cortellini et al., 2001). The randomized patient allocation was carried out by telephone on the day of the surgical intervention. The randomization list was based on permuted blocks with random block lengths. Patients were allocated in a 1:1 ratio. One experienced periodontist (M. F.) conducted all clinical measurements. He and the patients had no information about the group assignments (double‐blinding). IBM SPSS Statistics 26 (IBM) was used for statistical evaluation. Each patient contributed to the evaluation with one infrabony periodontal defect. Therefore, the patient was considered as a statistical unit. The defect‐related site with the highest PPD at baseline was used for the statistical evaluation of the clinical parameters BOP, PPD, RED, and CAL. The RBG_t0–t2_ was selected as the secondary outcome variable. A subgroup analysis was conducted for non‐containing defects defined by radiographic defect angle >22° (Tsitoura et al., 2004).

Multivariate linear regression analyses with backward elimination were performed to identify the variables with the greatest influence on the outcome variables ΔCAL_t0–t2_ and RBG_t0–t2_. Group was included a priori as an independent variable in the multivariate regression of both outcome variables. Patient‐ (age, gender, smoking habit, FMBS, HI) and defect‐related parameters (BOP, PPD_t0_, RED_t0_, defect angle, INFRA, INFRA_X‐ray_) were considered as further independent variables. Those that revealed the greatest influence on ΔCAL_t0–t2_ and RBG_t0–t2_, respectively, in previous univariate linear regression were selected for multivariate regression analysis. The general linear model (GLM) was applied to examine the temporal changes in PPD, RED, CAL, and RBG and to detect intragroup and intergroup differences. Further intergroup comparisons were performed using the *t* test for independent samples and the *χ* ^2^ test. All tests were performed two‐sided with a significance level of *α* = .05.

## 4 RESULTS

### 4.1 Study population

Patients were recruited from February 2014 to 2020. The test group included 20 patients (11 female, 9 male) with a mean age of 54.34 ± 13.40 years (range: 25.27–85.63 years) and the control group 20 patients (14 female, 6 male) with a mean age of 52.37 ± 15.24 years (range: 22.94–73.83 years). There were no significant differences between both groups regarding age, gender or smoking habits (test group: one smoker; control group: two smokers). There were five dropouts due to endodontic involvement (*n* = 1; test group), development of endo‐periodontal lesion (*n* = 1; test group), nonattendance to the follow‐up visits (*n* = 2; test group), and need for retreatment after abscess formation (*n* = 1; control group). Besides this, incomplete data sets were generated in two patients due to new pregnancy (*n* = 1; control group) and nonattendance to the follow‐up visit after 12 months (*n* = 1; control group). Thus, 35 patients were included in the statistical evaluation at t1, and 33 patients at t2.

### 4.2 Baseline defect characteristics

There was a comparable distribution of defects between maxilla (test group: *n* = 5; control group: *n* = 6) and mandible (test group: *n* = 15; control group: *n* = 14) in both groups (Table S1). Also one‐wall (test group: *n* = 4; control group: *n* = 4), two‐wall (test group: *n* = 13; control group: *n* = 14), and three‐wall defects (test group: *n* = 3; control group: *n* = 2) were comparably distributed. The remaining clinical and radiographic variables also showed no significant group‐dependent differences (Table 1).

**Table 1:** Baseline defect characteristics differentiated by group

Parameters		Test group (*n *= 20)	Control group (*n* = 20)	*p* value (95% CI)
		Mean ± SD	Mean ± SD	
Clinical	PPD (mm)	9.10 ± 1.48	9.10 ± 1.89	1.000 (−1.09; 1.09)
	RED (mm)	1.50 ± 1.32	1.60 ± 1.64	.833 (−1.05; .85)
	CAL (mm)	10.60 ± 1.79	10.70 ± 2.47	.884 (−1.48; 1.28)
Radiographic	Defect angle (degrees)	30.50 ± 7.94	27.98 ± 10.66	.402 (−3.50; 8.54)
	CEJ‐BD_X‐ray_ (mm)	10.34 ± 1.89	10.79 ± 2.86	.598 (−2.15; 1.26)
	INFRA_X‐ray_ (mm)	6.31 ± 1.75	7.37 ± 2.62	.142 (−2.48; .37)

Abbreviations: CAL, clinical attachment level; CEJ‐BD_X‐ray_, radiographic distance from cementoenamel junction to the bottom of the defect; INFRA_X‐ray_, radiographic infrabony component/distance from bone crest to bottom of the defect; PPD, probing pocket depth; *p* value (95% CI), *t* test for independent samples; RED, recession depth.

## 5 INTRAOPERATIVE MEASUREMENTS

No significant differences were found between the two groups for the intraoperative measurements. INFRA was 6.74 ± 1.89 mm in the test group and 7.30 ± 2.36 mm in the control group. The surgical intervention lasted on average 60.84 ± 16.84 min in the test group and 60.71 ± 10.99 min in the control group.

### 5.1 Clinical and radiographic outcome at 6‐months and 12‐months follow‐up visits

When considering the entire study population, no significant differences were found between the test and control group for the primary outcome variable ΔCAL_t0–t2_ and secondary outcome variable RBG_t0–t2_. Intragroup comparison using the GLM revealed that there was a significant PPD reduction (both groups: *p* < .001), RED increase (test group: *p* = .012; control group: *p* < .001), CAL gain (both groups: *p* < .001) and RBG (both groups: *p* < .001) in the two groups. The intergroup comparison showed that the temporal changes in RED differed significantly between the groups (GLM using “group” as a between‐subjects factor: *p* = .031). Regarding all patients, RED increase between t0 and t1 (ΔRED_t0–t1_) was significantly greater in the control group (*p* = .020; *t* test for independent samples). Further variables examined did not differ significantly in the intergroup comparison (Tables 2 and S2).

**Table 2:** Changes of PPD, RED, CAL, and RBG between baseline (t0) and the follow‐up visits 6 months (t1) and 12 months (t2) after surgery

Parameter		Δt0– t1			Δt0–t2		
		Test group (mean ± SD)	Control group (mean ± SD)	*p* value (95% CI)	Test group (mean ± SD)	Control group (mean ± SD)	*p* value (95% CI)
All patients	PPD (mm)	3.81 ± 1.52	3.63 ± 2.29	.789 (−1.18; 1.55)	4.38 ± 1.36	4.06 ± 2.38	.646 (−1.07; 1.71)
	RED (mm)	−0.50 ± 0.73	−1.26 ± 1.05	.020 (.13; 1.40)	−0.63 ± 0.72	−1.18 ± 1.24	.127 (−.17; 1.27)
	CAL (mm)	3.31 ± 1.58	2.37 ± 1.92	.126 (−.28; 2.17)	3.75 ± 1.24	2.88 ± 2.09	.160 (−.36; 2.10)
	RBG (mm)	2.40 ± 1.65	2.53 ± 2.08	.834 (−1.45; 1.17)	3.06 ± 1.74	3.27 ± 2.19	.768 (−1.63; 1.22)
Patients with defect angle >22°	PPD (mm)	3.62 ± 1.39	2.77 ± 1.24	.113 (−.22; 1.91)	4.31 ± 1.25	3.18 ± 1.60	.066 (−.08; 2.33)
	RED (mm)	−0.54 ± 0.78	−1.00 ± 0.71	.126 (−.14; 1.06)	−0.69 ± 0.75	−1.00 ± 1.00	.399 (−.43; 1.05)
	CAL (mm)	3.08 ± 1.38	1.77 ± 1.54	.032 (.12; 2.49)	3.62 ± 0.96	2.18 ± 1.83	.034 (.12; 2.75)
	RBG (mm)	2.21 ± 1.10	1.79 ± 0.95	.307 (−.41; 1.25)	2.98 ± 1.45	2.32 ± 0.64	.193 (−.36; 1.69)

*Note:* All patients: Δt0–t1: test group: *n* = 16, control group: *n* = 19; Δt0–t2: test group: *n* = 16; control group: *n* = 17. Patients with defect angle >22°: Δt0–t1: test group: *n* = 13, control group: *n* = 13; Δt0–t2: test group: *n* = 13; control group: *n* = 11.

Abbreviations: CAL, clinical attachment level; PPD, probing pocket depth; *p* value (95% CI), *t* test for independent samples; RBG, radiographic bone gain; RED, recession depth.

The COM showed that six defects (37.5%) of the test group and five defects (29.4%) of the control group had been successfully treated (relevant CAL gain, no residual pocket). Relevant CAL gain in combination with a residual pocket was observed in nine defects (56.3%) of the test group and five defects (29.4%) of the control group. Treatment failure was determined in one defect (6.3%) of the test group and five defects (29.4%) of the control group (Table 3).

**Table 3:** Composite outcome measure differentiated by group

		Residual PPD_t2_	
		≤4 mm	>4 mm
CAL gain _t0–t2_	≥3 mm	Test group: 6 (37.5%) control group: 5 (29.4%)	Test group: 9 (56.3%) control group: 5 (29.4%)
	<3 mm	Test group: 0 (0%) control group: 2 (11.8%)	Test group: 1 (6.3%) control group: 5 (29.4%)

Abbreviations: CAL, clinical attachment level; PPD, probing pocket depth.

We hypothesized that especially noncontaining defects with a large defect angle would benefit from the GTPT. Considering patients with baseline radiographic defect angle >22°, there was a significantly greater CAL gain in the test group than in the control group. Thus, ΔCAL_t0–t1_ was 3.08 ± 1.38 mm in the test group and 1.77 ± 1.54 mm in the control group (*p* = .032; *t* test for independent samples), and ΔCAL_t0–t2_ was 3.62 ± 0.96 mm in the test group and 2.18 ± 1.83 mm in the control group (*p* = .034; *t* test for independent samples). When evaluating patients with a baseline radiographic defect angle ≤22°, no significant differences between the two groups were found for any of the examined parameters (Table S4).

In the next step, multivariate linear regression with backward elimination was performed (Table 4). The previous univariate linear regression showed that the variables PPD_t0_, defect angle, and INFRA had a substantial influence on ΔCAL_t0–t2_ and were included in the multivariate linear regression. The variables PPD_t0_ (*p* = .001) and group (*p* = .064) remained in the last model and, thus, were identified as variables with the greatest influence on ΔCAL_t0–t2_. Furthermore, univariate linear regression revealed that the variables PPD_t0_, defect angle, and INFRA_X‐ray_ had the greatest influence on RBG_t0–t2_. Following backward elimination, INFRA_X‐ray_ (*p* = .003) and PPD_t0_ (*p* = .033) were identified as variables with the greatest influence on RBG_t0–t2_.

**Table 4:** Multivariate linear regression analysis with backward elimination using ΔCAL_t0–t2_ and RBG_t0–t2_ as dependent variables

Model		Included variables	Regression coefficient *β*	Significance	Adjusted *R* ^2^
Dependent variable: ΔCAL_t0–t2_	1	Group	−1.045	0.063	.277
		Defect angle	−.016	0.653	
		INFRA	.018	0.928	
		PPD_t0_	.524	0.039	
	2	Group	−1.042	0.058	.301
		Defect angle	−.017	0.600	
		PPD_t0_	.538	0.008	
	3	Group	−.974	0.064	.318
		PPD_t0_	.590	0.001	
Dependent variable: RBG_t0–t2_	1	Group	−.474	0.339	.565
		Defect angle	−.011	0.716	
		INFRA_X‐ray_	.461	0.005	
		PPD_t0_	.359	0.082	
	2	Group	−.442	0.357	.579
		INFRA_X‐ray_	.474	0.002	
		PPD_t0_	.380	0.052	
	3	INFRA_X‐ray_	.431	0.003	.581
		PPD_t0_	.411	0.033	

Abbreviations: ΔCAL_t0–t2_, clinical attachment level gain between baseline and 12 months after surgery; RBG_t0–t2_, radiographic bone gain between baseline and 12 months after surgery; INFRA, clinical infrabony component; INFRA_X‐ray_, radiographic infrabony component; PPD_t0_, probing pocket depth at baseline.

## 6 DISCUSSION

The hypothesis of the present study was that the GTPT would result in significantly more CAL gain than the double flap approach with resection of the defect‐filling granulation tissue 12 months after regenerative periodontal surgery. However, the study failed to find a significant difference between the test and control group for the primary outcome variable (ΔCAL_t0–t2_) (Table 2).

The intragroup comparison using the GLM revealed that significant PPD reduction (test group: 4.38 ± 1.36 mm; control group: 4.06 ± 2.38 mm), CAL gain (test group: 3.75 ± 1.24 mm; control group: 2.88 ± 2.09 mm), and RBG (test group: 3.06 ± 1.74 mm; control group: 3.27 ± 2.19 mm) were achieved in both groups 12 months after surgery. Recently, a meta‐analysis on the clinical performance of minimally invasive periodontal surgery (MIPS) for the treatment of infrabony defects was published (Clementini et al., 2019). The 18 studies included showed a PPD reduction of 4.24 mm, a RED increase of 0.44 mm, and a CAL gain of 3.89 mm, which is comparable to the results of our study. The lower CAL gain and higher RED increase observed in our study may be explained by the fact that the patient‐related factors HI and FMBS were not as good as in other studies, in which MIPS was applied (Cortellini et al., 2017; Ribeiro et al., 2011, 2013). Assessment of the treatment outcome using the COM revealed that relevant CAL gain ≥ 3 mm was achieved in 15 of 16 (93.8%) defects in the test group but only in 10 of 17 (58.8%) defects in the control group. In addition, there were five cases (29.4%) of treatment failure in the control group but only one case (6.3%) in the test group. This distribution shows how reliably GTPT generates relevant CAL gain and how rarely treatment failures occur.

Regarding baseline defect characteristics, advanced periodontal defects with a mean PPD >9 mm and a mean INFRA >6 mm were present in both groups. A systematic review has shown that deeper periodontal defects (>4 mm) are associated with more RBG than shallower defects (≤4 mm) (Nibali et al., 2021). Consistent with these findings, we observed by multivariate linear regression that PPD_t0_ and particularly INFRA_X‐ray_ had the greatest influence on RBG 12 months after surgery. This agrees with the results of other studies that also found a positive correlation between RBG and INFRA_X‐ray_ (Ilgenli et al., 2007; Liñares et al., 2006; Meyle et al., 2011). There is evidence that the defect morphology plays a crucial role in the outcome of regenerative periodontal therapy (Cortellini et al., 2008; Losada et al., 2017; Meyle et al., 2011). Comprehensive characterization of infrabony defects requires further factors such as the number of residual bone walls and the radiographic defect angle. In the present study, two residual bone walls were preserved in most of the defects (test group: *n* = 13; control group: *n* = 14). However, it was sometimes difficult to determine the number of residual bone walls beyond doubt during the intraoperative assessment. Thus, every one‐ or two‐wall defect has a more or less pronounced three‐wall component in the deeper area of the defect. To capture the heterogeneity of the defects in a more differentiated way, we decided to perform a separate analysis with subgroups determined by radiographic defect angle. A large defect angle is known to have a negative impact on the clinical and radiographic outcome (Eickholz et al., 2004; Losada et al., 2017). Accordingly, Tsitoura et al. reported that the probability of achieving CAL gain ≥ 4 mm is 2.5 times higher in defects with a defect angle ≤ 22° than in those with a defect angle ≥ 36° (Tsitoura et al., 2004). Our evaluation of patients with a defect angle >22° revealed that a significantly greater CAL gain was achieved in the test group. This difference was detectable after 6 and 12 months and was mainly a result of PPD reduction. The fact that the multivariate linear regression identified the variables PPD_t0_ and group as having the greatest influence on ΔCAL_t0–t2_ also supported this observation. This is consistent with the results reported by Eickholz et al. (2004), who investigated infrabony defects treated by the guided tissue regeneration technique. They also attested the initial PPD to be a significant predictor for CAL gain.

The rationale behind GTPT is that the use of bone substitutes can be avoided because the granulation tissue itself acts as soft tissue support. To achieve this goal, the granulation tissue should be mobilized in its entirety from the defect during the preparation of the mucoperiosteal flap. Special attention should be paid to placing the interdental incision on the bone crest (Figure 3), which sometimes requires bone sounding. Finally, the granulation tissue must be repositioned in its original position at the end of the surgical intervention. At this point, it is important to mention that both mobilization and repositioning of the soft tissue can be realized more easily the better the surgical area has been instrumented during the second step of periodontal therapy. One could assume that more time is needed for the surgical procedure of the GTPT. However, the duration of surgery was not significantly different between the two groups. In the present study, complete mobilization of the defect‐filling granulation tissue was not equally achieved in all defects. In general, it was more difficult to mobilize the entire granulation tissue in narrow three‐wall defects compared to wide one‐ and two‐wall defects. However, even if it was only possible to mobilize the coronal part of the granulation tissue, while the apical remained in the defect, the preservation and precise repositioning of the coronal part provided additional stability to the papillary soft tissue and facilitated the subsequent wound healing process.

Bone substitutes are frequently applied in regenerative therapy of advanced infrabony defects to prevent soft tissue collapse into the defect and, thus, preserve space for regeneration (Kao et al., 2015). There is conflicting data on whether EMD + graft material results in more CAL gain and greater PPD reduction than EMD alone. A recently published meta‐analysis looked at the treatment of infrabony defects using either EMD or EMD + graft material and differentiated the treatment outcome by flap design (Trombelli et al., 2021). They reported that EMD + graft material resulted in more CAL gain in minimally invasive variants (EMD: 3.69 mm; EMD + graft: 4.10 mm) and papilla preservation variants (EMD: 3.08 mm; EMD + graft: 3.65 mm) than EMD alone. However, other studies provide evidence that the use of EMD + graft material does not lead to more PPD reduction and greater CAL gain in the treatment of noncontaining defects (Hoffmann et al., 2016; Losada et al., 2017; Pietruska et al., 2012). These observations raise the question of whether it would have been better to use EMD + graft material in the control group instead of EMD alone.

Another significant limitation of the present study was that patients with containing defects (three‐wall defects, defect angle ≤ 22°) were also included. This was probably the main reason why no significant differences (apart from ΔRED_t0–t1_) were found between the test and control group when all study participants were considered. Another possible explanation could be that EMDs were used in both groups. Furthermore, the present study does not provide any information on whether periodontal regeneration or repair actually occurred during the wound healing process. Interestingly, there was a smaller RBG in the GTPT group compared to the control group, when all patients were considered. This was the case despite greater PPD reduction, greater CAL gain, and lower RED increase in the GTPT group. In contrast, patients with defect angle >22° tended to have a larger RBG in the test group. These contradictory data lead to the question of what happens to the granulation tissue during wound healing and what influence EMDs play in its maturation process. Animal studies could provide important information to clarify these questions.

## 7 CONCLUSIONS

We conclude that the GTPT may show its advantages over the conventional technique especially in advanced cases characterized by unfavorable defect morphology, namely few residual bone walls and large defect angle. Conversely, if sufficient residual bone walls and/or small defect angles are still present, removal of the granulation tissue does not represent a disadvantage for the regenerative healing process.

## CONFLICT OF INTERESTS

The authors declare that there are no conflict of interests.

## ETHICS STATEMENT

The present study has been approved by the Institutional Review Board (Ethics Committee of Hannover Medical School, reference number: 5556) and all participants signed an informed consent according to the Declaration of Helsinki.

## AUTHOR CONTRIBUTIONS

Knut Adam conceived the study, performed the surgical procedures, analyzed and interpreted the data, and drafted the manuscript. Hüsamettin Günay participated in the study design, supervised the surgical interventions, and critically reviewed the manuscript. Bernhard Vaske conducted the sample size calculation and assisted in performing the statistical analysis. Marco Flohr performed the measurements of the clinical parameters and critically reviewed the manuscript. Ingmar Staufenbiel conceived the study, performed the surgical procedures, and critically reviewed the manuscript. All authors read and approved the final version of the manuscript.

## Supporting information

Supporting information.

Supporting information.

Supporting information.

Supporting information.

Video Clip S4.

## Data Availability

Data generated during the study are not publicly available but can be obtained from the corresponding author on reasonable request.
