# Supplementary material for: The granulation tissue preservation technique in regenerative periodontal surgery—a randomized controlled clinical trial
Source: Clin Exp Dent Res. 2022 Jan 11;8(1):9–19. doi: 10.1002/cre2.532 (PMC8874108; doi:10.1002/cre2.532)
Supplement: Supplementary file 1 — Supporting information. [file CRE2-8-9-s004.docx]

Table S1: Distribution of infrabony periodontal defects by group, jaw and tooth type

| Tooth type | Test group | | Control group | | Total |
| --- | --- | --- | --- | --- | --- |
|  | Maxilla | Mandible | Maxilla | Mandible |  |
| Incisor | 0 | 0 | 2 | 1 | 3 |
| Canine | 1 | 2 | 1 | 2 | 6 |
| Premolar | 3 | 2 | 3 | 0 | 8 |
| Molar | 1 | 11 | 0 | 11 | 23 |
| Total | 5 | 15 | 6 | 14 | 40 |
