# Supplementary material for: The granulation tissue preservation technique in regenerative periodontal surgery—a randomized controlled clinical trial
Source: Clin Exp Dent Res. 2022 Jan 11;8(1):9–19. doi: 10.1002/cre2.532 (PMC8874108; doi:10.1002/cre2.532)
Supplement: Supplementary file 2 — Supporting information. [file CRE2-8-9-s005.docx]

Table S2: Gingival inflammation and plaque control differentiated by group

|  | | Test group  Mean ± SD | Control group  Mean ± SD | p-value (95% CI) |
| --- | --- | --- | --- | --- |
| FMBS  [%] | t0 | 20.16 ± 9.18 | 16.68 ± 9.21 | 0.240 (-2.41; 9.36) |
|  | t1 | 18.68 ± 8.00 | 20.45 ± 9.03 | 0.549 (-7.68; 4.16) |
|  | t2 | 21.50 ± 6.41 | 23.10 ± 9.20 | 0.565 (-7.22; 4.02) |
| HI  [%] | t0 | 57.06 ± 14.31 | 54.36 ± 15.21 | 0.567 (-6.75; 12.15) |
|  | t1 | 52. 94 ± 16.05 | 49.14 ± 12.92 | 0.443 (-6.16; 13.76) |
|  | t2 | 47.91 ± 14.31 | 46.85 ± 9.28 | 0.805 (-7.64; 9.75) |

FMBS: full mouth bleeding score; HI: hygiene index; t0: baseline; t1: 6 months after surgery; t2: 12 months after surgery; p-value: t-test for independent samples.
