# Supplementary material for: The granulation tissue preservation technique in regenerative periodontal surgery—a randomized controlled clinical trial
Source: Clin Exp Dent Res. 2022 Jan 11;8(1):9–19. doi: 10.1002/cre2.532 (PMC8874108; doi:10.1002/cre2.532)
Supplement: Supplementary file 3 — Supporting information. [file CRE2-8-9-s003.docx]

Table S3: Early healing index (EHI) at treated sites 1, 2 and 3 weeks after surgery

| EHI | Test group | | | Control group | | |
| --- | --- | --- | --- | --- | --- | --- |
|  | 1 week  n (%) | 2 weeks  n (%) | 3 weeks  n (%) | 1 week  n (%) | 2 weeks  n (%) | 3 weeks  n (%) |
| 1 | 12 (63.2) | 16 (84.2) | 15 (78.9) | 10 (50.0) | 14 (70.0) | 15 (75.0) |
| 2 | 6 (31.6) | 1 (5.3) | 3 (15.8) | 8 (40.0) | 4 (20.0) | 2 (10.0) |
| 3 | 1 (5.3) | 2 (10.5) |  | 1 (5.0) |  | 1 (5.0) |
| 4 |  |  | 1 (5.3) | 1 (5.0) | 2 (10.0) | 2 (10.0) |
| 5 |  |  |  |  |  |  |
