# Supplementary material for: The granulation tissue preservation technique in regenerative periodontal surgery—a randomized controlled clinical trial
Source: Clin Exp Dent Res. 2022 Jan 11;8(1):9–19. doi: 10.1002/cre2.532 (PMC8874108; doi:10.1002/cre2.532)
Supplement: Supplementary file 4 — Supporting information. [file CRE2-8-9-s002.docx]

Table S4: Changes of PPD, RED, CAL and RBG in patients with a baseline radiographic defect angle ≤ 22 degrees

| Parameter | Δ t0 – t1 | | | Δ t0 – t2 | | |
| --- | --- | --- | --- | --- | --- | --- |
|  | Test group (n=3)  Mean ± SD | Control group (n=6)  Mean ± SD | p-value (95% CI) | Test group (n=3)  Mean ± SD | Control group (n=6)  Mean ± SD | p-value (95% CI) |
| PPD [mm] | 4.67 ± 2.08 | 5.50 ± 3.02 | 0.685 (-5.48; 3.82) | 4.67 ± 2.08 | 5.67 ± 2.88 | 0.613 (-5.47; 3.47) |
| RED [mm] | -0.33 ± 0.58 | -1.83 ± 1.47 | 0.142 (-0.64; 3.64) | -0.33 ± 0.58 | -1.50 ± 1.64 | 0.284 (-1.21; 3.55) |
| CAL [mm] | 4.33 ± 2.31 | 3.67 ± 2.16 | 0.682 (-3.02; 4.35) | 4.33 ± 2.31 | 4.17 ± 2.04 | 0.915 (-3.38; 3.71) |
| RBG [mm] | 3.18 ± 3.45 | 4.13 ± 3.00 | 0.682 (-6.19; 4.29) | 3.44 ± 3.14 | 4.87 ± 2.95 | 0.523 (-6.46; 3.60) |

PPD: probing pocket depth; RED: recession depth; CAL: clinical attachment level; RBG: radiographic bone gain; p-value (95% CI): t-test for independent samples.
